# Risk of skeletal related events among elderly prostate cancer patients by site of metastasis at diagnosis

**DOI:** 10.1002/cam4.914

**Published:** 2016-10-11

**Authors:** Arif Hussain, Abdalla Aly, C. Daniel Mullins, Yi Qian, Jorge Arellano, Eberechukwu Onukwugha

**Affiliations:** ^1^ University of MarylandSchool of MedicineMarlene and Stewart Greenebaum Cancer Center BaltimoreBaltimoreMaryland; ^2^ Veterans Affairs Medical CenterBaltimoreMaryland; ^3^ Pharmerit InternationalBethesdaMaryland; ^4^ Department of Pharmaceutical Health Services ResearchUniversity of MarylandSchool of PharmacyBaltimoreMaryland; ^5^ Amgen Inc.Thousand OaksCalifornia

**Keywords:** Prostate cancer, site of metastasis, skeletal‐related events

## Abstract

The purpose of this study was to estimate the risk of developing skeletal‐related events (SREs) based on site of metastasis at diagnosis and identify other predictors of developing SREs among metastatic prostate cancer patients. We conducted a retrospective cohort study using linked SEER (Surveillance, Epidemiology, and End Results) and Medicare data and identified men over the age of 65 with incident metastatic prostate cancer diagnosed during 2005–2009. SREs included radiation (RAD), pathological fractures (PF), bone surgery (BS), and spinal cord compression (SCC). The association between site of metastasis at diagnosis and SRE was examined using a Cox proportional hazards model that accounts for death as a competing risk. Among 4404 men (median age: 79 years) with incident metastatic prostate cancer, 44% experienced SREs at a median of 9.6 months post diagnosis. Compared to bone metastasis only, our model showed that patients were significantly less likely to develop SREs if they had LN‐only metastasis at diagnosis (Sub‐Hazard Ratio [SHR] 0.56; 95% Confidence Interval [CI]: 0.43–0.72) or unknown site of metastasis (SHR: 0.79; CI: 0.64–0.97). Other predictors of reduced SRE risk were age 80+ years (SHR: 0.83; CI: 0.75–0.91), non‐Hispanic Black (SHR: 0.77; CI: 0.65–0.90), or being diagnosed in year 2009 (SHR: 0.85; CI: 0.72–0.99). Patients were significantly more likely to develop SREs if they received androgen deprivation therapy (SHR: 1.73; CI: 1.48–2.02) or had Gleason score 8–10 disease (SHR: 0.79; CI: 0.64–0.97). Compared to patients who present with bone metastasis only at diagnosis, patients presenting with other metastatic sites have similar risk of developing SREs, with the exception of those presenting with lymph node only metastasis who have a significantly reduced risk of SREs.

## Introduction

The prognosis of patients diagnosed with Stage 4 prostate cancer is significantly impacted by the presence or absence of metastasis at diagnosis. Patients diagnosed with M1 (metastatic) disease have worse survival compared to patients with stage 4 (S4) M0 disease. A large study reported that the most commonly encountered metastatic sites at diagnosis were bone (84%), distant lymph nodes (10.6%), liver (10.2%), and thorax (9.1%), while 18.4% of patients had more than one organ involved 1.

Two recent studies demonstrate that the site of metastasis impacts survival rates. In a study that used Surveillance, Epidemiology, and End Results (SEER) data from 1991 to 2009, patients with visceral metastasis had poor survival compared to patients with lymph node involvement only 2. The same study estimated that the median overall survival for lymph node, bone, visceral, and bone plus lymph node metastasis at diagnosis were 43, 24, 16, and 14 months respectively. In a meta‐analysis that pooled data from 5 phase III randomized clinical trials (RCTs), overall survival for castrate‐resistant patients with lymph node only, liver ± bone, lung ± bone, bone ± lymph node, and other visceral metastasis (adrenal, brain) were 27.0, 12.1, 16.5, 20.3, and 14.4 months 3.

Several population‐based observational studies have shown that bone metastasis is associated with greater risk of skeletal complications, commonly referred to as skeletal‐related events (SREs), including pathologic fracture (PF), spinal cord compression (SCC), bone palliative radiotherapy (RAD), and bone surgery (BS), and contribute significantly to the burden of prostate cancer 4, 5, 6. Zoledronic acid and, more recently denosumab, have been approved by the Food and Drug Administration since they delay onset of SREs in patients with bone metastasis 7, 8. While it is known that patients with bone metastasis at diagnosis are at high‐risk of SREs, there is limited information on the impact of other sites of metastasis at presentation on the risk of SREs. The purpose of this study was to estimate the risk of developing an SRE among S4M1 patients presenting with various sites of metastasis at diagnosis and to identify patient factors that correlated with the risk of developing SRE.

## Patients and Methods

### Data source

We used linked Surveillance, Epidemiology, and End Results (SEER)‐Medicare datasets to study the relationship between the site of metastasis at diagnosis and risk of developing an SRE during follow‐up. The SEER‐Medicare database links information from the National Cancer Institute's SEER cancer registries and Medicare claims data from the Centers for Medicare and Medicaid Services. The SEER program collects cancer incidence and mortality rates from 17 tumor registries across the U.S. covering 28% of the U.S. population 9. Medicare claims provide information on health care services which are provided to and covered for Medicare beneficiaries from the time of Medicare eligibility until death.

### Study cohort

**Figure 1:**
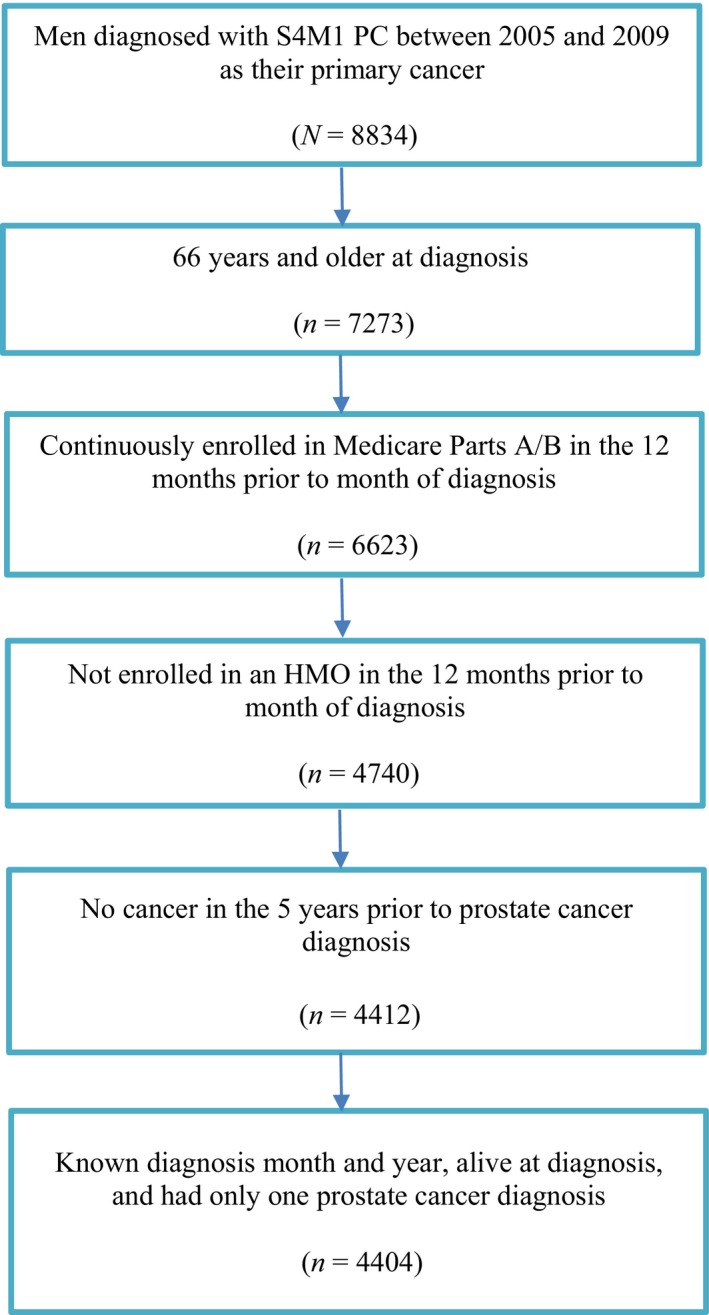
Cohort identification flow chart.

This study used a retrospective cohort study design to identify patients with prostate cancer (SEER code 54). Information on the specific sites of distant metastasis in patients with M1 prostate cancer at diagnosis became available in the SEER registries of 2004 onwards, (using the derived American Joint Committee on Cancer stage grouping system, 6th edition) whereas such detailed staging information for M1 patients was not available prior to 2004 10. Thus, to obtain more accurate staging data this study included men aged 66 or older in SEER who were diagnosed with incident cases of M1 prostate cancer between 2005 and 2009. The 2004 cohort was excluded since it represented the first year of extracting the more ‘granular’ incident staging information from patient medical records, and hence was potentially more prone to discrepancies in documentation than during the subsequent years when more experience in extracting such data was gained. The follow‐up period ended on December 31, 2010, or earlier if patients enrolled in a health maintenance organization or dis‐enrolled in Medicare Parts A and B or died during this time period. Patients were required to have continuous enrollment in Medicare Parts A/B in the year prior to diagnosis in order to assess baseline Charlson Comorbidity Index (CCI) in the year prior to diagnosis. Patients were excluded from the final sample if they had history of cancer in the 5 years prior to diagnosis, if their diagnosis month or year was unknown, or if they received a postmortem prostate cancer diagnosis (Fig. 1).

### Exposure, covariate, and endpoint definition

We used the ‘CS Mets at DX’ measure to identify the location of distant metastasis at diagnosis among the 2005 to 2009 SEER cohort (http://web2.facs.org/cstage0204/prostate/Prostate_hal.html). SREs were identified using Medicare claims, including the International Classification of Diseases 9th version Clinical Modification and the Healthcare Common Procedure Coding System that indicated SCC, PF, BS, or RAD (Table**** 1), which was previously published 11. Covariates used in the model included demographic variables (age at diagnosis, race/ethnicity, Census location), clinical variables (CCI, performance status proxies), prostate cancer variables (Gleason score at diagnosis), and treatment (androgen deprivation therapy receipt).

**Table 1:** ICD‐9 Codes and HCPCS codes used for identifying skeletal‐related events (SRE) measures

Spinal cord compression	
ICD‐9	3369, 7211, 7214, 72141, 72142, 72191, 7227, 72270, 72271 and 72273
HCPCS	63050, 63051, 22551, 22552,, 63064, 63066, 61343, s2348, 63075‐8, s2350, s2351, 63195, 63197, 63199, 63001, 63003, 63005, 63011, 63015, 63016, 63017, 63170, 63012, 63045, 63046, 63047, 63048, 63040, 63042, 63043, 63044, 63020, 63030, 63035, 22224, 22222, 22214, 22212, 22207, 22206, 0274t, 0275t, c9729, 0202t, 22865, 0164t, 0094t, 0097t, 63057, 63056, 63055, 63081, 63082, 63087, 63088, 63101, 63102, 63103, 63090, 63091, 63086 and 63085
Pathologic fractures	
ICD‐9	7331, 73311, 73312, 73313, 73314, 73315, 73316, and 73319
HCPCS	8202, 8208, 8210, 8212, 73311, 8120, 8122, 8124, 73312, 8130, 8132, 8134, 8138, 73316, 8230, 8232, 8238, 73313, 805, 806, 8200, 7331, 73310, 73319, 800, 807, 8080, 8082, 8084, 8088, 8100, 8240, 8242, 80701, 80702, 80703, 80704, 80705, 80706, 80707, 80708, 80709, 80841, 80842, 80843, and 80849
Trauma/nonroutine falls/accidents	
ICD‐9	819, 828, 851, 852, 853, 854, 860, 861, 862, 863, 864, 865, 866, 867, 868, 869, 8074, 9584, 80712, 80713, 80714, 80715, 80716, 80717, 80718, 80719, E800‐E848, E881, E882, E883, E884.0, E884.1, E884.5, E885.0, E885.1, E885.2, E885.3, E885.4, E886.0, E886.9, E888.0, and E888.1
Bone palliative radiotherapy	
ICD‐9	9223, 9224, 9229, 9230, 9231, 9232, and 9239
HCPCS	A9600, A9604, A9605, C9401, G0173, G0174, G0243, G0251, G0339, G0340, J3005, 0073T, 61793, 61796, 61797, 61798, 63620, 63621,77371, 77372, 77373, 77401, 77402, 77403, 77404, 77406, 77407, 77408, 77409, 77411, 77412, 77413, 77414, 77416, 77418, 79005, 79101, 79200, 79300, 79400, 79403, 79440, 79445, and 79999
Bone surgery	
ICD‐9	7815, 7845, 7855, 7915, 7925, 7935, 7995, 7812, 7842, 7852, 7911, 7921, 7931, 7991, 7813, 7843, 7853, 7912, 7922, 7932, 7992, 7817, 7847, 7857, 7916, 7926, 7936, 7996, 0353, 8102, 8103, 8104, 8105, 8106, 8107, 8108, 7810, 7811, 7816, 7819, 7840, 7841, 7846, 7849, 7850, 7851, 7856, 7859, 7910, 7919, 7920, 7929, 7930, 7939, 7990, and 7999
HCPCS	27187. 27235. 27236. 27244. 27245. 27248. 27269. 27495. 27506. 27507. 27509. 27511. 27513. 27514. 23615. 23616. 23630. 24498. 24515, 24516. 24538, 24545, 24546, 24566, 24575, 24579, 24582, 24586, 24587, 24635, 24665, 24666, 24685, 25490, 25491, 25492, 25515, 25525, 25526, 25545, 25606, 25607, 25608, 25609, 27535, 27536, 27745, 27756, 27758, 27759, 27766, 27769, 27784, 27792, 27826, 27827, 22325, 22326, 22327, 22328, 22520, 22521, 22522, 22532, 22533, 22534, 22548, 22550, 22554, 22555, 22556, 22558, 22565, 22585, 22590, 22595, 22600, 22610, 22612, 22614, 22615, 22625, 22630, 22632, 20982, 23490, 23515, 23585, 27215, 27216, 27217, 27218, 27226, 27227, 27228, 27524, 27540, 22523, 22524, 22525, 22526, 22527, 25574, and 25575

### Statistical analysis

We examined the bivariate distributions between sociodemographic, clinical, and prostate cancer‐specific factors as they relate to SRE status. Sub‐Hazard Ratios (SHR) of experiencing a SRE were derived using the inverse probability of treatment weighted (IPTW) Cox proportional hazards model that accounted for deaths as a competing risk and adjusted for age, race/ethnicity, androgen deprivation therapy receipt, comorbidities, performance status, Gleason score, and year of diagnosis. The IPTW was obtained in a two‐step process. We first estimated the propensity score using a logistic regression modeling the probability of androgen deprivation therapy (ADT) receipt as the dependent variable. Then the inverse of the propensity score was used to weight the sample in a Cox proportional hazards model. IPTW was important since men who received ADT were expected to be systematically different from those who did not receive ADT, therefore adjusting for selection bias. Additionally, ADT was included in the Cox proportional hazards model since men who received ADT (even after mimicking randomization) were expected to have more SREs compared to men who did not receive ADT, thus adjusting for confounding bias due to ADT. Applying IPTW and Cox proportional hazards model is a form of doubly robust estimation that protects against mismodeling 12. Since site of metastasis influences the hazard of death, a competing risks framework was preferred because site of metastasis may have no direct influence on the hazard of SRE but can be significantly associated with cumulative probability of SRE. A cumulative incidence plot was generated for each site of metastasis based on the competing risks model. In our model, those who were lost to follow‐up (HMO enrollment, Medicare Parts A/B disenrollment, or end of follow‐up on December 31, 2010) were censored. All statistical analyses were performed using SAS software package (version 9.3, SAS Institute, Cary, NC) and Stata software package (version 13*,* Stata*,* College Station, TX).

## Results

### Study sample characteristics

Among 4404 patients (mean follow up: 16.6 months) diagnosed with incident metastatic prostate cancer, 1135 (25.8%) did not receive ADT, which is similar to what has been reported in prior SEER‐Medicare studies 13, 14. Non‐Hispanic Whites, and those with lower CCI and higher Gleason scores were more likely to experience SREs (*P* < 0.05). Table**** 2 shows the distribution of several of the available sociodemographic, clinical, and tumor‐related characteristics in SEER, as categorized by SRE status at follow‐up.

**Table 2:** Demographic and clinical characteristics among M1 prostate cancer men diagnosed from 2005 to 2009, by skeletal‐related events status (*N* = 4404)

	Any skeletal‐related event (*N* = 4404)				
	No (*n* = 2473)		Yes (*n* = 1931)		*P* value
	*N*	%	*N*	%	
Age					0.06
66–70	416	17	356	18	
71–75	457	18	390	20	
76–80	499	20	402	21	
80 +	1101	45	783	41	
Race/Ethnicity	1824	74	1530	79	<0.01*
Non‐Hispanic White					
Non‐Hispanic Black	368	15	200	10	
Hispanic	160	6	106	6	
Other	121	5	95	5	
SEER census location					
Northeast	476	19	391	20	0.16
South	487	20	329	17	
North Central	353	14	282	15	
West	1157	47	929	48	
Married	1427	58	1139	59	0.39
Urban residence	2160	87	1732	90	0.01*
Charlson comorbidity index					
0	1254	51	1059	55	<0.01*
1	462	18	396	21	
2	239	10	161	8	
3+	241	10	175	9	
Missing	277	11	140	7	
Androgen deprivation therapy	1652	67	1617	84	<0.01*
Prediagnosis poor performance function	706	29	501	26	0.05
High PSA at baseline	2099	85	1664	86	0.23*
Poorly differentiated tumor	1457	59	1172	61	0.23
Gleason score					
2–6	99	4	49	2	<0.01*
7	319	13	212	11	
8–10	997	40	884	46	
Not done/unknown	1058	43	786	41	
Year of diagnosis					
2005	482	19	466	24	<0.01*
2006	517	21	420	22	
2007	460	19	363	19	
2008	503	20	370	19	
2009	511	21	312	16	

*Significant at the *P* = 0.05 level.

### Association between metastatic site and SRE

**Figure 2:**
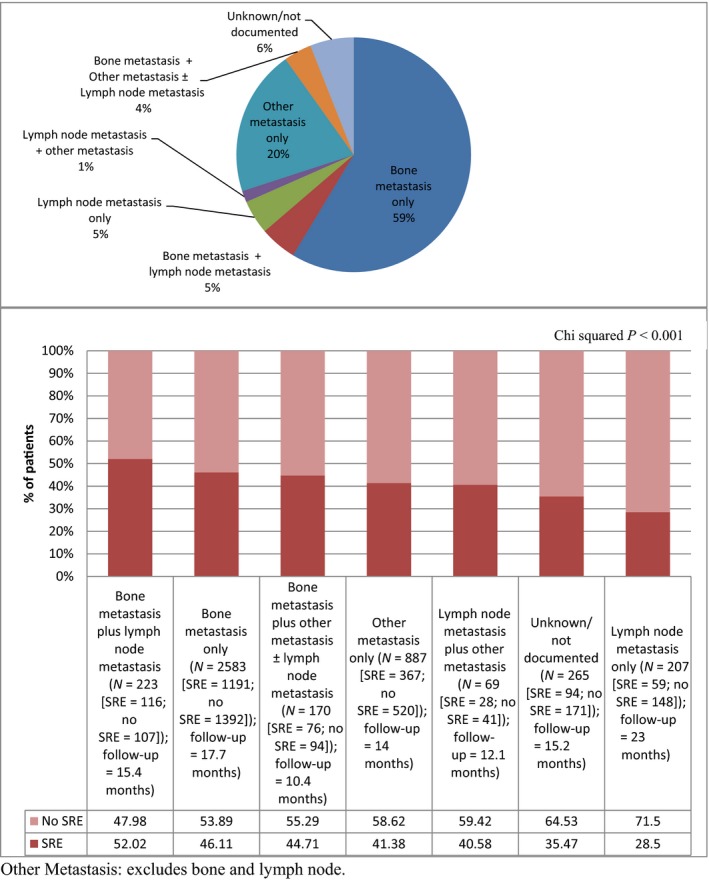
Proportion of patients with the various metastatic sites at presentation and their skeletal‐related events distribution.

The distribution of various sites of metastasis among the incident cases of M1 prostate cancer patients in the final sample is shown in Figure 2. Staging information was not available in 6% of patients in this cohort. The bone, with or without lymph node and/or ‘other’ (including visceral) sites of involvement represents the most common site of distant spread, with metastasis to bone only occurring in 59% of patients at initial presentation and to bone ± other sites in 68%. Twenty percent of the sample presented with metastasis to sites other than the bone or lymph node (designated as ‘other only’ sites which would include visceral organs), whereas only a minority of patients (4.7%) had lymph node only metastasis at initial presentation. Overall, 10% of men had metastasis to two or more ‘organ’ sites (i.e., bone, lymph node and/or ‘other’ sites) at diagnosis. On average, 44% of the final sample developed a SRE during follow‐up. The proportion of patients with the different sites of metastasis at initial presentation who developed a subsequent SRE during the follow‐up period is shown in Figure 2; this ranged from 29% (lymph node metastasis only) to 52% (bone and lymph node metastasis). There was a statistically significant difference between SRE rates across the seven metastatic sites (*P *<* *0.001).

**Figure 3:**
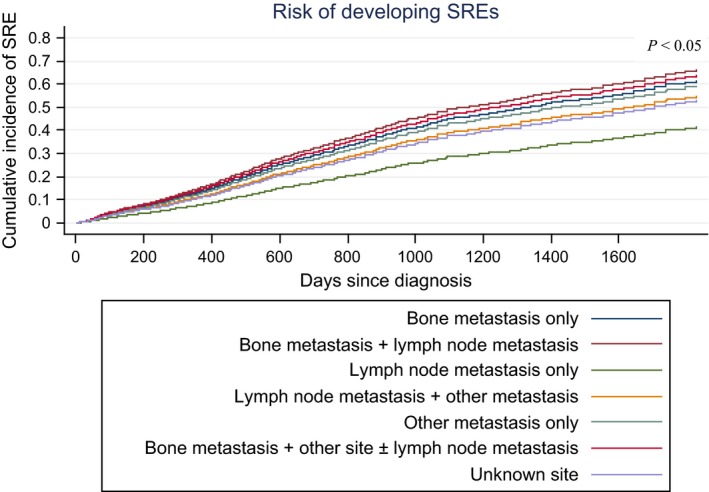
Inverse probability weighted and covariate adjusted cumulative incidence of experiencing an skeletal‐related events according to metastatic site at diagnosis.

In the Cox proportional hazards model that accounts for death as a competing risk, it is apparent that among the prostate cancer men with different sites of metastasis those with lymph node only involvement at diagnosis were significantly less likely to develop a SRE during follow‐up compared to patients presenting with bone only metastasis at diagnosis (Sub‐Hazard Ratio (SHR): 0.56; 95% Confidence Interval (CI): 0.43–0.72). Patients with ‘other’ site only, bone plus lymph node, bone plus ‘other’ site ± lymph node, and lymph node plus ‘other’ site were as likely as patients with bone metastasis only to develop SREs. Lastly, although the relevant extent of disease information was missing in the ‘unknown site of metastasis’ patient group, this group was also less likely to develop SREs (SHR: 0.79; 95% CI: 0.64–0.97) compared to the bone metastasis only group (Table 3), perhaps in part reflecting that it may be more akin to the lymph node only population in terms of clinical behavior. Using the same model we have produced a cumulative incidence of SRE plot that shows the probability of developing SREs among various sites of metastasis. The probability of developing SREs within 3 years of diagnosis with Stage IV M1 prostate cancer among patients with various sites of metastasis were: lymph node only (29%), unknown site (36%), lymph node + other site (39%), other site (43%), bone only (45%), bone +other ± lymph node (47%), and bone + lymph node (50%).

**Table 3:** Covariate adjusted SHR for skeletal related events among patients diagnosed with M1 prostate cancer diagnosed from 2005 to 2009

	Any SRE (*N* = 4404)	
	SHR	95% CI
Metastatic Site	Reference	
BM Only		
LN Only	0.56*	(0.43, 0.72)
Other Only	0.94	(0.83, 1.06)
BM + LN	1.13	(0.92, 1.40)
BM + Other ± LN	1.06	(0.82, 1.37)
LN + Other	0.83	(0.54, 1.26)
Unknown	0.79*	(0.64, 0.97)
Age	Reference	
≤80		
80+	0.83*	(0.75, 0.91)
Race/ethnicity	Reference	
Non‐hispanic white		
Non‐hispanic black	0.77*	(0.65,0.90)
Hispanic	0.86	(0.70,1.06)
Other	0.90	(0.72,1.11)
Androgen deprivation therapy	1.73*	(1.48,2.02)
Charlson comorbidity index	Reference	
0		
1	1.04	(0.92,1.17)
2	0.89	(0.74,1.07)
3+	0.95	(0.80,1.14)
Missing	0.86	(0.71,1.03)
Prediagnosis poor performance function	1.18	(0.97,1.45)
Gleason score	Reference	
2–6		
7	1.19	(0.88,1.61)
8–10	1.50*	(1.13,1.98)
Not done/unknown	1.66*	(1.25,2.21)
Year of diagnosis	Reference	
2005		
2006	0.91	(0.79,1.04)
2007	0.91	(0.78,1.05)
2008	0.87	(0.75,1.00)
2009	0.85*	(0.72,0.99)

SRE, skeletal related event; BM, bone metastasis; Other, Other metastasis (excludes LN and bone); LN, Lymph node metastasis; SHR, sub‐hazard ratios. *Significant at the *P* = 0.05 level. Poor performance function indicates a claim for a walking aid or wheelchair use. We also controlled for SEER location. We also controlled for census location and marital status (not shown).

### Other predictors of developing SREs

In the multivariable Cox proportional hazards model, patients were significantly less likely to develop SREs if they were over 80 years of age (SHR: 0.83; 95% CI: 0.75–0.91), were of non‐Hispanic Black ethnicity (SHR: 0.77; 95% CI: 0.65–0.90), or were diagnosed in year 2009 (SHR: 0.85; 95% CI: 0.72–0.99). On the other hand, patients were significantly more likely to develop SREs if they received androgen deprivation therapy (SHR: 1.73; 95% CI: 1.48–2.02), or had a Gleason score of 8–10 (SHR: 1.5; 95% CI: 1.13–1.98) (Table**** 3). Interestingly, the overall prevalence of SREs decreased over time from 2005 to 2009 (*P* < 0.01).

### SRE subtypes

Several statistically significant differences are noteworthy regarding the frequency of the different subtypes of SRE (i.e., RAD, BS, SCC, PF) with respect to certain covariates such as metastatic site, age, race, diagnosis year, and ADT receipt (Table**** 4 **)**. Radiation was statistically less likely among men who had lymph node only metastasis at diagnosis, were 80 years of age or older, were diagnosed with MI prostate cancer in year 2009 compared to the earlier years, or did not receive ADT for their prostate cancer. Bone surgery was statistically less likely among African American men, and those men who were diagnosed with prostate cancer in 2009. Pathologic fractures were less likely among African Americans, those less than age 80 or those who did not receive ADT. On the other hand, no significant differences with respect to spinal cord compression were found among the different covariates examined except for those M1 patients who did not receive ADT (this latter group had lower incidence of SCC) (Table**** 4).

**Table 4:** Distribution of skeletal‐related events subtype by select covariates

	Any SRE	RAD	BS	SCC	PF
Metastatic site					
LN only (*n* = 207)	28.50	16.43	NR	NR	9.18
BM only (*n* = 2,583)	46.11	24.12	2.79	2.32	16.88
Other only (*n* = 287)	41.38	21.08	NR	NR	15.56
BM + LN (*n* = 223)	52.02	28.25	NR	NR	17.04
BM + Other ± LN (*n* = 170)	44.71	24.12	NR	NR	14.71
LN + Other (*n* = 69)	40.58	20.29	NR	NR	17.39
Unknown (*n* = 265)	35.47	17.36	NR	NR	13.58
*P* value	<0.001	0.008*	0.33	0.65	0.11
Age					
66–80 (*n* = 1372)	45.56	26.19	2.34	2.74	14.29
80 + (*n* = 1101)	41.56	18.47	2.87	1.96	18.26
*P* value	0.008*	<0.01*	0.28	0.1	<0.01*
Race					
Non‐Hispanic White (*n* = 1824)	45.62	23.17	3.01	2.59	16.85
African American (*n* = 368)	35.21	20.95	NR	NR	11.09
Hispanic (*n* = 160)	39.85	21.43	NR	NR	15.79
Other (*n* = 121)	43.98	25.46	NR	NR	15.74
*P* value	<0.001*	0.48	0.008*	0.32	0.007*
Diagnosis year					
2005 (*n* = 482)	49.16	25.53	3.69	3.38	16.56
2006 (*n* = 517)	44.82	23.91	2.99	2.45	15.47
2007 (*n* = 460)	44.11	23.94	2.67	NR	15.19
2008 (*n* = 503)	42.38	21.08	NR	NR	17.41
2009 (*n* = 511)	37.91	19.56	NR	NR	15.19
*P* value	<0.001*	0.02*	0.02*	0.19	0.64
Charlson comorbidity index					
Zero (*n* = 1254)	46.09	25.78	2.12	2.67	15.52
1 (*n* = 462)	47.13	22.25	3.50	NR	19.38
2 (*n* = 239)	40.94	21.26	NR	NR	15.22
3+ (*n* = 241)	43.52	19.43	NR	NR	18.91
*P* value	0.18	0.01*	0.07	0.48	0.04*
ADT					
No (*n* = 1135)	27.67	8.81	3.35	1.50	14.01
Yes (*n* = 3269)	49.46	27.78	2.29	2.72	16.67
*P* value	<0.001*	<0.01*	0.05	0.02*	0.035*

NR, Not reported per data use agreement with NCI. PF, pathological fractures; SCC, spinal cord compression.

*Significant at the *P* = 0.05 level.

## Discussion

Metastasis to the bone is a common occurrence in men with advanced prostate cancer, and is associated with significant morbidity and mortality 15, 16, 17, 18. One approach to understanding the clinically relevant consequences of bone metastasis is to study what has been defined as SREs. This includes certain interventions such as RAD and BS, or certain clinical events such as PF and SCC, that can occur among patients with bone metastasis. Prospective clinical trials in patients with established bone metastasis have provided important information about SREs in cancer patients 15, 19, 20. In an effort to better understand the occurrence patterns and impact of PF, SCC, RAD, and BS in a broader prostate cancer population than what is typically defined in controlled clinical trials, we used the SEER‐Medicare dataset to conduct the present analysis in a large cohort of men with stage IV M1 prostate cancer. This study took advantage of the fact that since 2004 onwards more detailed staging information on M1 patients is being captured in SEER (M1a, M1b, M1c; i.e., sites of metastasis at diagnosis).

In contrast with most prior studies where SREs have been studied in men with bone metastasis 4, 5, 15, 19, 20, 21, our study is unique in that we evaluated patients with different sites of metastasis, including those without bone metastasis at diagnosis, to determine the risk of developing SREs (as determined from claims data) among these different subgroups of prostate cancer patients. Using this approach, the present work documents that SREs can occur in all subcategories of M1 patients, although those with lymph node‐only metastasis at presentation are significantly less likely to experience SREs compared to the other subgroups (Fig. 2, Table 3). One possible reason why men with lymph node metastasis only at diagnosis have less SREs may be due to their lower likelihood of developing subsequent bone metastasis. However, since we were not able to identify bone metastasis after diagnosis, this cannot be confirmed from the present data.

In addition to the initial sites of metastasis, we found age, ethnicity, year of cancer diagnosis and ADT receipt, can also affect the risk of developing SREs among the M1 prostate cancer cohort (Table 4). Regarding age we found that the overall lower incidence of SREs in the 80+ year old group is primarily due to the lower use of radiation amongst these patients compared to the 66–80 year old age group. Amongst African Americans, lower risk of developing PF and lower use of BS (perhaps a consequence of lower PF) account for their overall lower incidence of SREs compared to the other ethnic groups. This observation is not inconsistent with the known lower risk of fractures in African Americans compared to European Americans, perhaps reflecting inherent differences in their respective skeletal physiology 22.

Another interesting observation relates to the use of ADT. ADT is the mainstay of treatment, and in fact represents the first line of treatment for M1 prostate cancer patients. Despite this, remarkably, 25.8% of the M1 cohort did not have claims for ADT receipt, a figure that is not inconsistent with what has been reported previously by others 10, 11. Claims reflecting all four SRE subcomponents are significantly less in the non‐ADT group than in the corresponding ADT group (Table**** 4). The duration of follow‐up for the non‐ADT group is also considerably less than for the ADT group (4.9 vs. 19.5 months). Whether non‐ADT patients receive lesser extent of medical services in general, as reflected by not getting a standard therapy (ADT) for their cancer in the first place and having significantly less follow‐up compared to ADT patients, and whether such factors in part contribute to lower SRE‐related claims across all SRE subtypes among this group, is not altogether clear but will require further study.

This study has several limitations. First, the codes used to define SREs have not been validated and are subject to further research. A Danish study validated the ICD‐10 coding of bone metastasis and SREs in prostate cancer and found that the sensitivity of ICD‐10 codes ranged from 44% to 55% and specificity ranged from 94% to 100% 23. Second, there is no billing code for SREs which makes it harder to directly identify SREs, especially radiation to bone. The inability to differentiate receipt of radiation to the prostate gland from radiation to the bone will result in overestimating the prevalence of radiation. However, by only assessing S4M1 patients, we believe that the majority of our sample receiving radiation is using it for bone palliation. Third, this study did not include younger patients diagnosed with incident S4M1 or elderly patients who were initially diagnosed with nonmetastatic disease but developed bone metastasis during follow‐up.

In conclusion, this study documents risk of SREs among elderly metastatic prostate cancer patients, irrespective of whether patients had bone metastasis at diagnosis. Although we cannot determine from these data if such patients go on to develop bone metastasis over time, these results do provide important evidence for patients and oncologists concerning SRE risk among all metastatic patients. We also identified several factors such as age and race/ethnicity that can modify the risk of SREs among metastatic prostate cancer patients. The slight decrease in SREs over time is promising. Better prevention and management of SREs can help to minimize their impact on men with advanced prostate cancer.

## Conflict of Interest

None declared.
